# Pemafibrate, a New Selective PPARα Modulator: Drug Concept and Its Clinical Applications for Dyslipidemia and Metabolic Diseases

**DOI:** 10.1007/s11883-020-0823-5

**Published:** 2020-01-23

**Authors:** Shizuya Yamashita, Daisaku Masuda, Yuji Matsuzawa

**Affiliations:** 1 Department of Cardiology, Rinku General Medical Center, Izumisano, Osaka, 598-8577 Japan; 2 Sumitomo Hospital, Osaka, Japan

**Keywords:** Peroxisome proliferator-activated receptor alpha (PPARα), Selective PPAR alpha modulator (SPPARMα), Pemafibrate, Triglycerides, Dyslipidemia

## Abstract

**Purpose of Review:**

Reduction of serum low-density lipoprotein cholesterol (LDL-C) levels by statins, ezetimibe and proprotein convertase subtilisin/kexin type 9 (PCSK9) inhibitors has been shown to significantly reduce cardiovascular events risk. However, fasting and postprandial hypertriglyceridemia as well as reduced high-density lipoprotein cholesterol (HDL-C) remain as residual risk factors of atherosclerotic cardiovascular diseases (ASCVD). To treat patients with hypertriglyceridemia and/or low HDL-C, drugs such as fibrates, nicotinic acids, and n-3 polyunsaturated fatty acids have been used. However, fibrates were demonstrated to cause side effects such as liver dysfunction and increase in creatinine levels, and thus large-scale clinical trials of fibrates have shown negative results for prevention of ASCVD. The failure could be attributed to their low selectivity and potency for binding to peroxisome proliferator-activated receptor (PPAR) α. To resolve these issues, the concept of selective PPARα modulator (SPPARMα) with a superior balance of efficacy and safety has been proposed and pemafibrate (K-877) has been developed.

**Recent Findings:**

Pemafibrate, one of SPPARMsα, was synthesized by Kowa Company, Ltd. for better efficiency and safety. Clinical trials in Japan have established the superiority of pemafibrate on effects on serum triglycerides (TG) reduction and HDL-C elevation as well safety. Although available fibrates showed worsening of liver and kidney function test values, pemafibrate indicated improved liver function test values and was less likely to increase serum creatinine or decrease estimated glomerular filtration rate (eGFR). Very few drug-drug interactions were observed even when used concomitantly with statins. Furthermore, pemafibrate is metabolized in the liver and excreted into the bile, while many of available fibrates are mainly excreted from the kidney. Therefore, pemafibrate can be used safely even in patients with impaired renal function since there is no significant increase in its blood concentration. A large-scale trial of pemafibrate, PROMINENT, for dyslipidemic patients with type 2 diabetes is ongoing.

**Summary:**

Pemafibrate is one of novel SPPARMsα and has superior benefit-risk balance compared to conventional fibrates and can be applicable for patients for whom the usage of existing fibrates is difficult such as those who are taking statins or patients with renal dysfunction. In the current review, all the recent data on pemafibrate will be summarized.

## Introduction

Fibrates were developed in the 1950s based upon the discovery of phenylethyl acetate from agricultural chemical ingredients which were found to reduce serum lipids [1]. Clofibrate was the first fibrate which was classified as a lipid-lowering agent. Later, several fibrates were developed and shown to enhance the proliferation of peroxisome in mice, but the mechanism of action of fibrates remained unknown for many years. Currently available fibrates were developed without definitive knowledge of their specific mechanism of action. However, fibrates were later demonstrated to act on PPARα and elicit their biological effects such as reduction of serum triglycerides (TG) levels and increase in serum HDL-C levels [2, 3]. Although the activation of PPARα by fibrates markedly improved serum lipid levels, a variety of off-target effects such as abnormal test values suggesting liver and kidney dysfunctions was observed, which were difficult to be overcome.

Large-scale clinical trials of fibrates for prevention of cardiovascular (CV) events were subsequently conducted. In the Helsinki Heart Study (HHS) [4] and the Veterans Affairs High-Density Lipoprotein Cholesterol Intervention Trial (VA-HIT) [5], administration of gemfibrozil significantly reduced CV event rate, the primary endpoints of the trials. However, significant drug-drug interactions between gemfibrozil and cerivastatin resulted in a very high incidence of rhabdomyolysis in patients [6]. Furthermore, in subsequent trials, including Benzafibrate Infarction Prevention (BIP) study (using bezafibrate alone) [7], Fenofibrate Intervention and Event Lowering in Diabetes (FIELD) study (using fenofibrate alone) [8], and the Action to Control Cardiovascular Risk in Diabetes (ACCORD)-lipid study (using fenofibrate on top of simvastatin) [9], primary endpoints were statistically negative and the clinical efficacy of fibrates on CV events was questioned.

The meta-analysis of fibrates [10, 11] demonstrated significant reductions of CV event rate, and the significant reduction of CV event risk was shown for each test, particularly in the *post-hoc* analysis of the subclasses of patients with or without statin who had atherogenic dyslipidemia (high serum TG and low HDL-C levels) [12]. The ACCORDION study, a passively extended follow-up observation of the ACCORD study, indicated a long-term continuous benefit of fenofibrate [13]. Similarly, in the BIP study, a persistent benefit in reducing mortality was demonstrated especially in patients with baseline hypertriglyceridemia [14]. Other meta-analyses in both primary and secondary prevention of CV events showed supportive evidences [15, 16]. The meta-analyses of statins by the Cholesterol Treatment Trialists’ (CTT) Collaboration [17–19] showed that the administration of statins significantly reduced the total mortality rate by ~ 10%. In contrast, a significant decrease in the total mortality rate upon administration of fibrates could not be demonstrated [10, 11]. The off-target effects of fibrates as mentioned above may have offset their efficacy. The lack of a significant mortality benefit by fibrates has led many doctors to consider them as a second choice.

In this context, the development of novel therapeutic strategies for atherogenic dyslipidemia in patients associated with diabetes, metabolic syndrome, obesity, and/or ASCVD was urgently demanded. Selective peroxisome proliferator-activated receptor (PPAR) α modulators (SPPARMα) may provide a promising future for the management of atherogenic dyslipidemia and atherosclerosis as well as other metabolic abnormalities [20].

### Subtypes of Peroxisome Proliferator-Activated Receptors (PPARs)

PPARs are one of the nuclear hormone receptors that bind to DNA as a heterodimer with retinoid X receptor (RXR). This heterodimer recognizes specific DNA sequences in and around target genes called PPAR response elements (PPREs). Many genes carry response elements for PPARs. After the structure of PPAR was clarified, three PPAR isoforms (PPARα, PPARγ, and PPARδ) have been identified, each of which is encoded by a separate gene [21]. The PPARα subtype is abundant in highly active metabolic tissues such as the liver, heart, muscle, kidney, brown adipose tissue, and vascular wall cells, including endothelial cells, smooth muscle cells, and macrophages. To the contrary, PPARγ is expressed mainly in white and brown adipose tissues, large intestine, and macrophages. PPARδ (also called PPARβ) is expressed ubiquitously.

Endogenous ligands such as free fatty acids, prostaglandins, leukotrienes, or synthetic PPAR agonists such as fibrates for PPARα and glitazones for PPARγ, respectively, bind to the ligand-binding domain forming heterodimer with ligand-activated RXR [22–25]. This binding causes the conformational change which influences cofactor affinity and thus results in transactivation or trans-repression of target genes. While PPAR is transactivated, the activated PPAR binds to PPRE in the upstream of target genes and the PPAR complex becomes transcriptionally active with involvement of cofactors [20, 26–29].

### Pleiotropic Functions of PPARα

PPARα is crucially involved in metabolic homeostasis [30]. PPARα regulates lipid and lipoprotein metabolism since it is associated with the transcription of genes that are involved in the reduction of serum TG and increase in HDL-C [2]. Activation of PPARα increases the production of lipoprotein lipase (LPL) and apolipoprotein (apo) A-V, while it decreases the plasma levels of apo C-III that inhibits LPL activity, thereby enhancing the catabolism of TG-rich lipoproteins and reducing serum TG levels [31–34]. PPARα activation also upregulates the expression of genes involved in the β-oxidation pathways. Fatty acids levels in the liver are decreased through enhanced β-oxidation and increased expression of hepatic acyl-CoA synthase (ACS) [35]. Thus, hepatic production of very-low-density lipoprotein (VLDL) particles is attenuated [29].

In addition to the effects on TG-rich lipoproteins, the activation of PPARα reduces the number of atherogenic small dense low-density lipoprotein (LDL) particles [36]. VLDL enriched with apo C-III interact better with cholesteryl ester transfer protein (CETP), increasing the exchange of TG from VLDL to LDL, and these TG-rich LDL particles become small dense LDL after lipolysis of TG by hepatic lipase (HL) [37]. Thus, the activation of PPARα reduces small dense LDL particles by (1) producing apo C-III-poor VLDL particles that interacts less well CETP and (2) increasing the formation of large buoyant LDL that has a high affinity to and are easily taken up by LDL receptor.

The activation of PPARα also enhances HDL synthesis by increasing the expression of apo A-I and A-II, both of which are major components of HDL [38]. Increased LPL activity enhances the lipolysis of TG-rich lipoproteins thereby increasing the sources of phospholipids on HDL particles [39]. The activation of PPARα also accelerates reverse cholesterol transport (RCT) via increased expression of ABCA1 and ABCG1 transporters involved in cholesterol efflux from macrophages [29, 40]. PPARα activation also increases the expression of scavenger receptor class B type I (SR-BI) in the liver which is involved in the selective uptake of cholesteryl ester by the liver and enhance RCT [41].

Furthermore, PPARα activation may also be involved in glucose homeostasis regulation, inhibition of inflammation and thrombogenesis, and improved vascular function [26, 28, 29, 42]. However, the underlying mechanisms for these effects in humans have not been clarified yet. PPARɑ activation improves abnormal lipid and/or glucose metabolism, possibly leading to vascular protection against atherothrombosis by down-regulation of proinflammatory genes in monocytes/macrophages [26, 27]. Thus, pharmacological targeting of PPARα activation may be one of the important strategies for patients with diabetes, metabolic syndrome, obesity, and atherosclerotic CV diseases.

### Novel Concept and Rationale for SPPARMα

Based upon these backgrounds, a novel concept of selective PPARα modulator (SPPARMα) was originally proposed by Fruchart [43, 44••]. The principle of SPPARMα action was illustrated in these reviews [43, 44••]. The concept of SPPARMα has an analogy to selective estrogen receptor modulators (SERMs) [45]. This concept of SERMs was based upon the paradigm of tamoxifen. This drug is the first estrogen receptor modulator and has anti-estrogenic activity in the mammary gland and a partial pro-estrogenic activity in the uterus and bone. However, long-term usage of tamoxifen increased the incidence of uterine cancer. Thus, a second-generation SERM, raloxifene, with tissue-specific activity was developed. Following the concept of SERM, SPPARMα with tissue-specific and targeted gene-selective activities has been developed.

PPARs are known to have a large lipid-binding pocket which can encompass endogenous ligands. When some ligands bind to PPARs, a conformational change occurs which leads to recruitment of co-activators, resulting in tissue-specific and gene-selective effects. Importantly, PPAR ligands share cofactors leading to a shared biological response; however, there exist some differences in cofactor selectivity, leading to differing responses. Ligand-specific cofactors and the unique receptor–cofactor binding profile of the ligand may be a crucial determinant of the specificity and potency of receptor binding, resulting in modulation of gene- and tissue-selective effects. Modulation of the receptor–cofactor binding profile of the PPAR ligand may improve desirable biological effects by transactivation of desirable target genes and reduce known adverse effects of the PPAR ligand by trans-repression of undesirable genes. This is the rationale for the development of SPPARMs. The candidate materials should be screened and identified from those which can differentially induce a unique receptor–cofactor binding profile, provide improved efficacy, and avoid unwanted side effects [43].

### Development of a Novel SPPARMα, Pemafibrate

Ligands with various structures such as free fatty acids and fibrates can bind to PPARα. The binding of each ligand to PPARα induces downstream ligand-specific structural changes and subsequent responses based upon association with ligand-specific cofactors. SPPARMα comes from the concept of drugs that can selectively regulate the transcription of PPARα target genes involved in beneficial actions, but not unbeneficial ones. Therefore, SPPARMα may have a better benefit-risk balance compared to the PPARα agonists such as available fibrates.

**Fig. 1:**
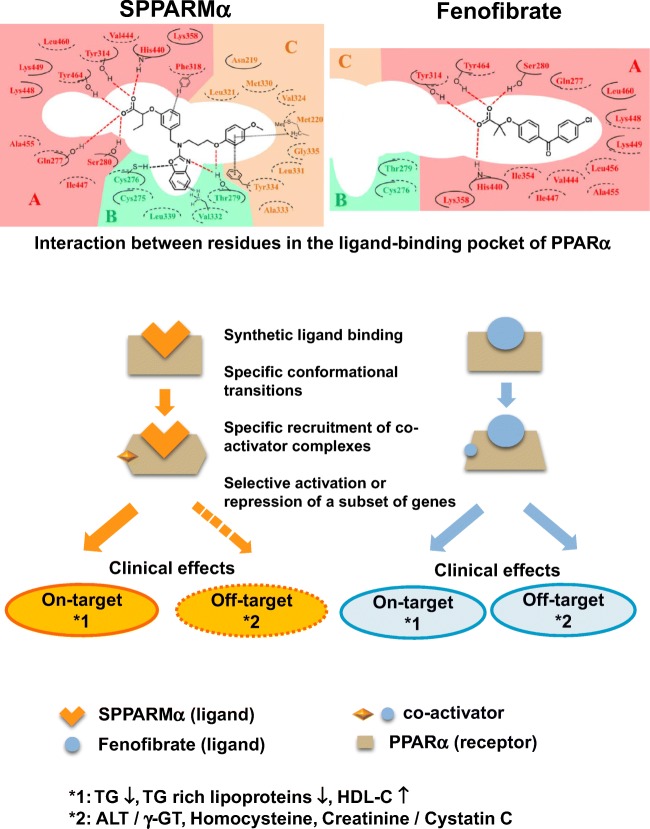
Structural differences in the interaction of pemafibrate and fenofibrate with PPARα and the concept of SPPARMα**.** The binding affinity of pemafibrate to PPARα is markedly increased compared with that of fenofibrate because pemafibrate has many interaction sites in regions A, B, and C of PPARα. The specific binding of pemafibrate with a high affinity results in specific conformational transitions of PPARα, recruiting specific co-activator complexes. Thus, pemafibrate has specific effects on target genes, but not off-target genes. In contrast, fenofibrate has specific effects on target as well as off-target genes. Reproduced from ref [47••]. Partially modified from refs [43] and [48]

Kowa Company, Ltd., in Japan screened more than 1500 compounds and identified several candidates, including pemafibrate, as agonists which possess a very potent PPARα activity and very high PPARα selectivity. Three candidate compounds (R-24, R-35, and R-36) were identified. From these, Kowa Company finally selected R-36 for development and R-36 was finally named pemafibrate (K-877, Parmodia^®^ tablet). Pemafibrate has an acidic region in its structure as demonstrated in other PPARα agonists. However, the addition of unique benzoxazole and phenoxyalkyl side-chains has contributed to the greatly enhanced PPARα activity and selectivity [46] (Fig. 1). To construct pemafibrate, a 2-aminobenzoxazole ring was inserted into the existing fibric acid skeleton, the length of carbon chain was modified and a phenoxyalkyl group was also introduced to enable synthesis of this drug as a highly active and selective PPARα agonist [46]. The PPARα activation by pemafibrate was > 2500 times stronger than fenofibric acid, the active form of fenofibrate. Pemafibrate is an extremely selective PPARα agonist (subtype selectivity > 5000-fold for PPARγ and > 11,000-fold for PPARδ, respectively) [49, 50].

Pemafibrate was demonstrated to have an equivalent or stronger TG-lowering activity compared to fenofibric acid in rats without increasing liver weight [46]. Transcriptome analysis of pemafibrate-regulated genes in primary human hepatocytes and the mouse liver has indicated that the induced and suppressed gene groups clearly differed between pemafibrate and fenofibrate [51]. Different from conventional fibrates, pemafibrate has a Y-shaped structure (Fig. 1). The ligand-binding domain in PPARα is Y-shaped. The Y-shaped pemafibrate is bound firmly to the whole cavity area like a locked key, followed by specific conformational changes in the structure of PPARα because of the strong interaction. Then, a new region of PPARα is exposed. This new region binds with PGC-1α, one of the PPARα co-activators, which results in a strong activation of PPARα. Recent studies have indicated that in addition to the portion of fibric acid, which is common to the conventional fibrates, a Y-shaped structure with suitably arranged aminobenzoxazole and dimethoxybenzene, is the ideal structure for SPPARMα [47••]. Pemafibrate has been proven to possess an ideal structure that corresponds to the concept of SPPARMα.

In June 2018, pemafibrate was launched in Japan prior to the rest of the world. The Japan Atherosclerosis Society, together with overseas evaluations, has positioned pemafibrate as a drug classified into the SPPARMα category, which is completely different from conventional fibrates [52]. Recently, a consensus statement from the International Atherosclerosis Society (IAS) and the Residual Risk Reduction Initiative (R3i) Foundation on the concept of SPPARMα has been published [53••]. Pemafibrate is thus expected to have a superior benefit-risk balance compared to the conventional fibrates.

### Pharmacokinetics and Metabolism of Pemafibrate

In vitro studies using hepatocytes from rats, monkeys, and humans analyzed the metabolic profiles and pharmacokinetics of pemafibrate [54]. Hepatocytes from rats, monkeys and humans could all transform pemafibrate to its demethylated form (M1). The bioavailability of pemafibrate was 15% in Sprague–Dawley rats and 87% in cynomolgus monkeys, respectively, after a single oral administration of 1 mg/kg pemafibrate. Unmetabolized pemafibrate was predominant in rat plasma, which accounted for 29% of the area under the curve of total radioactivity. In monkey plasma, the major circulating metabolites were M2/3 (dearylated/dicarboxylic acid forms, 15%), M4 (*N*-dealkylated form, 21%) and M5 (benzylic oxidative form, 9%), but pemafibrate was the minor form (3%). The metabolite profile of pemafibrate in plasma was different between rats and monkeys, and the latter could be a suitable animal model for further pharmacokinetic studies of pemafibrate in humans. In humans, pemafibrate is predominantly metabolized by the liver and not by kidney.

### Preclinical and Animal Studies

In preclinical studies, pemafibrate was demonstrated to have more robust effects of TG reduction and HDL-C elevation than fenofibrate. In Sprague–Dawley rats, pemafibrate inhibited VLDL secretion and enhanced TG clearance by activation of LPL [49]. VLDL and its remnants can be taken up by the liver via LDL receptor and VLDL receptor [55]. Fenofibrate [56] and pemafibrate [51] were reported to enhance the expression of VLDL receptor, which may result in the enhanced catabolism of VLDL and its remnants.

Pemafibrate (1 mg/kg) increased HDL-C levels more markedly than fenofibrate (100 mg/kg) in transgenic apo E2 mice and enhanced cholesterol efflux from macrophages. It reduced the atherosclerotic lesions in the aorta of apo E2KI mice [57], showed a strong anti-inflammatory effect, and attenuated atherosclerosis after mechanical injury [57–59].

Pemafibrate suppressed postprandial hyperlipidemia by inhibiting the mRNA expression of intestinal cholesterol transporter NPC1L1 in small intestine mucosa in mice fed a high-fat diet [60•, 61]. Thus, pemafibrate attenuates postprandial hyperlipidemia by suppression of chylomicron synthesis and secretion via inhibition of cholesterol absorption via NPC1L1 as well as PPARα activation in the small intestines.

Pemafibrate upregulates the expressions of genes related to β-oxidation of fatty acids, thereby inhibiting the secretion of VLDL from the liver [49, 61]. Fibroblast growth factor 21 (FGF21) is involved in β-oxidation of fatty acids, and its expression is regulated by PPARα [62, 63]. FGF21 also reduces VLDL secretion from the liver via regulation of fatty acids uptake by adipose tissue [64]. Pemafibrate increases the serum levels and tissue expression of FGF21 [49, 61, 65•]. The reduction of serum TG and VLDL levels by pemafibrate may partly be attributed to the upregulation of FGF21 via PPARα.

LPL catalyzes the hydrolysis of TG in chylomicrons secreted from the intestine and VLDL secreted from the liver. LPL is also involved in the particle uptake of VLDL by the liver as a ligand. The administration of pemafibrate in animal models increased LPL activity and thereby accelerated the catabolism of chylomicrons and VLDL [60•, 61]. The enhanced LPL activity by pemafibrate is due to the increase in LPL synthesis via activation of PPARα. Pemafibrate reduces serum levels of apo C-III and Angptl3, both of which are inhibitors of LPL [49]. Apo A-V accelerates plasma TG hydrolysis by LPL [66], but pemafibrate may not affect apo A-V levels despite PPARα activation [61]. Preclinical data indicate that pemafibrate has beneficial effects on atherogenic dyslipidemia, inflammation, and atherosclerosis by modulating PPARα-mediated gene expressions.

### Clinical Trials of Pemafibrate

#### Effect of Pemafibrate on Lipid, Lipoprotein, and Apoprotein Metabolism

Fasting hypertriglyceridemia is one of the risk factors of CAD and is based upon an increase in either of TG-rich lipoproteins, such as chylomicrons derived from small intestines, VLDL derived from the liver, and their TG-hydrolyzed remnant lipoproteins such as chylomicron remnants and VLDL remnants (IDL). Remnant lipoproteins have been demonstrated to be proatherogenic [67, 68]. Increased TG-rich lipoproteins are often associated with the presence of atherogenic small dense LDL and reduction of HDL-C. Pemafibrate significantly reduces remnant lipoprotein cholesterol (RemL-C), non-HDL-C, apo B, apo B-48, and apo C-III levels. Regarding the size of lipoprotein subfractions after pemafibrate treatment analyzed by gel permeation high-performance liquid chromatography (GP-HPLC), pemafibrate dose-dependently reduced small LDL particles and increased small HDL particles [69].

Postprandial hypertriglyceridemia is known as a risk status for CAD due to increases in atherogenic chylomicron remnants. To assess the effect on postprandial hypertriglyceridemia, pemafibrate (0.4 mg/day) was administered for 4 weeks in dyslipidemic patients and meal tolerance test was performed before and after treatment. A marked reduction of both fasting and non-fasting serum levels of TG, RemL-C, and apo B-48 was demonstrated [70•]. The incremental AUC for postprandial TG level was significantly reduced after pemafibrate treatment, suggesting that it improves postprandial hypertriglyceridemia. Similar data were obtained in diabetic patients treated with pemafibrate [71••].

**Fig. 2:**
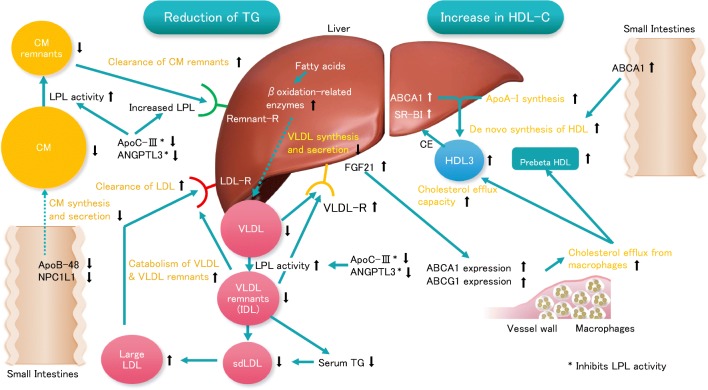
Molecular mechanisms for the favorable effects of pemafibrate on lipoprotein metabolism and reverse cholesterol transport. Abbreviations: ABCA1, ATP-binding cassette transporter A1; Angptl3, angiopoietin-like protein 3; LPL, lipoprotein lipase; Remnant-R, Remnant receptor; SR-BI, scavenger receptor class B type I; VLDL-R, VLDL receptor

The particle number in each lipoprotein subclass can also be calculated by GP-HPLC [72]. Pemafibrate reduced the number of atherogenic small LDL particles, while it increased that of antiatherogenic small HDL particles [73]. Reduced cholesterol efflux capacity (CEC) of HDL was shown to correlate with the presence of CAD [74] and the risk of CV events [75]. CEC is now recognized as one of the new CAD risk factors and smaller-sized HDL has greater CEC [76]. The effect of pemafibrate 0.4 mg/day for 4 weeks on CEC of HDL was evaluated in patients with dyslipidemia [70•]. The levels of HDL-C, HDL3-C, preβ1HDL, and apoA-1 were significantly increased by administration of pemafibrate. The HDL obtained from patients treated with pemafibrate demonstrated a significantly increased CEC from macrophages compared with HDL obtained from patients given placebo. Pemafibrate also increased the levels of FGF21, which is known to increase the expression of ATP-binding cassette transporters A1 and G1 (ABCA1 and ABCG1) involved in the cholesterol efflux from macrophages [49, 51, 57, 61, 70•, 77]. The possible molecular mechanisms for the favorable effects of pemafibrate on lipoprotein metabolism and reverse cholesterol transport are illustrated in Fig. 2.

#### Co-Administration of Pemafibrate with Statins

Co-administration of fibrates with statins was reported to increase the incidence of rhabdomyolysis, particularly in patients with impaired kidney function. Especially, high incidence of rhabdomyolysis occurred in patients administered a combination of gemfibrozil and cerivastatin, and thus cerivastatin disappeared from the market. This was a typical case of drug-drug interaction [6]. The mechanism of rhabdomyolysis on co-administration of these drugs may be gemfibrozil-mediated inhibition of cerivastatin metabolism, resulting in an increase in cerivastatin concentration.

Drug-drug interaction of pemafibrate with high doses of various statins (pravastatin, simvastatin, fluvastatin, atorvastatin, pitavastatin, or rosuvastatin) was investigated in healthy male volunteers [78]. Pemafibrate given to volunteers in combination with various statins gave no major changes in Cmax and AUC of pemafibrate or in those of any statins. The serum concentration of simvastatin and its open acid form was slightly decreased, but HMG-CoA reductase inhibitory activity was maintained. Thus, pemafibrate does not show any drug interactions with statins. Arai et al. reported a combined treatment of pemafibrate with statins in two clinical studies [79•]. Pemafibrate was administered for 12 weeks in patients with hypertriglyceridemia taking pitavastatin. It reduced the fasting TG values by 6.9% in the placebo group, 46.1% in the 0.1 mg/day pemafibrate group, 53.4% in the 0.2 mg/day group, and 52.0% in the 0.4 mg/day group, respectively. In the long-term study, 0.2–0.4 mg/day pemafibrate was administered for 24 weeks to patients with hypertriglyceridemia treated with any statins mentioned above. Pemafibrate showed consistent reduction of TG and no significant increase in adverse effects in association with pemafibrate was demonstrated. Even when co-administrated with statins, pemafibrate improved liver function test values. Serum levels of creatinine slightly increased and eGFR slightly decreased by pemafibrate administration although these changes were clinically negligible.

#### Comparisons of Pemafibrate with Fibrates

The first three clinical trials in Japan showed a higher efficacy of pemafibrate compared with fenofibrate [69, 80•, 81•]. The TG-lowering effect of 0.4 mg/day pemafibrate was more potent than that of 100 mg/day (80 mg/day with tablet conversion) and 106.6 mg/day fenofibrate, respectively, and was similar to 200 mg/day fenofibrate (160 mg/day with tablet conversion). The incidence of adverse events in patients treated with pemafibrate was almost similar to that treated with placebo, which was shown much lower than fenofibrate. Especially, the incidence of adverse events related to kidney and liver function was extremely rare. Fibrates are known to worsen kidney function test values, including serum creatinine and cystatin C levels, and estimated glomerular filtration rate (eGFR) [82–84]. While fenofibrate administration increased serum levels of creatinine and cystatin C and decreased eGFR during the treatment, these changes were much smaller in the pemafibrate-treated group. Fibrates are known to increase liver function test values, and the mechanism of this action is attributed to activation of PPARα [85]. Fibrates such as fenofibrate are known to worsen liver function test values such as alanine aminotransferase (ALT) and gamma-glutamyl transferase (γ-GT). However, treatment with pemafibrate was not associated with an increase, but rather a decrease of these values. These changes in renal and liver function test values after treatment are one of the characteristic features of SPPARMα, pemafibrate.

Recent meta-analysis compared the efficacy and safety of pemafibrate in patients with dyslipidemia in comparison with fenofibrate [86••]. The reduction of serum TG and non-HDL-C levels and increase in HDL-C by pemafibrate were comparable to those by fenofibrate. The homeostasis model assessment for insulin resistance (HOMA-IR) was also improved by pemafibrate, suggesting that insulin resistance is attenuated by this drug. Pemafibrate also demonstrated a significant decrease in hepatobiliary enzyme activity compared with the placebo and fenofibrate. Total adverse events were significantly less in the pemafibrate group than in the fenofibrate group. In contrast, the LDL-C level was significantly higher in the pemafibrate group than in the placebo and fenofibrate groups.

#### Pemafibrate for Patients with Chronic Kidney Disease (CKD)

Patients with CKD, especially those under hemodialysis, do not usually have high levels of LDL-C, but increased TG and decreased HDL-C levels [87]. Statin trials such as 4D [88] and AURORA [89] failed to demonstrate the efficacy to reduce CV events in patients with CKD. In SHARP trial [90], non-statin drug ezetimibe (intestinal cholesterol transporter inhibitor) showed a significant reduction in CV events. It is crucial to prevent CV events in patients with CKD, especially those under hemodialysis. Their typical phenotypes of lipoprotein abnormality are hypertriglyceridemia, increased remnants, and low HDL-C.

It has been very difficult to treat CKD patients with dyslipidemia since most of conventional fibrates except for clinofibrate were excreted from kidney. However, a SPPARMα, pemafibrate, is mainly metabolized in the liver and almost exclusively excreted from the liver, with only 14.5% excretion in the urine [91]. The main metabolites of pemafibrate in plasma are benzyl oxidized oxidant and a mixture of glucuronic acid conjugate and *N*-dealkylated dicarboxylic acid [92]. Excretion rate of unmetabolized pemafibrate into urine is < 0.5%, and almost all the metabolized compounds excreted in the urine have no PPARα agonist activity.

The exposure of pemafibrate was demonstrated not dependent on severity of renal dysfunction [93]. Administration of pemafibrate for a longer period was confirmed to be also effective and safe in patients with dyslipidemia, including patients with impaired kidney function. No major increase in blood pemafibrate concentrations was observed even after administering repeated dosage [94]. Fenofibrate and bezafibrate are current fibrates used in common. However, blood concentrations of these drugs with renal excretion properties are increased in patients with impaired renal function. Based upon its metabolic route, pemafibrate can be administered safely even to patients with CKD. Pemafibrate might have an improved benefit-risk balance and may be beneficial for patients with a limited ability to use conventional fibrates.

### Other Non-lipid-Related Effects of Pemafibrate

#### Effects on Glucose Metabolism, Insulin Resistance, and Obesity

Long-term administration for 24 weeks of pemafibrate in hypertriglyceridemic patients with type 2 diabetes showed similar effects on lipids and lipoproteins, but fasting blood glucose and insulin levels were significantly reduced by pemafibrate in comparison with placebo [71••]. Fifty-two week data from the PROVIDE study after administration of pemafibrate in hypertriglyceridemic patients with type 2 diabetes were also reported [95••]. The levels of TG and non-HDL cholesterol were stably decreased, while HDL-C levels were increased with administration of pemafibrate over 52 weeks. Pemafibrate was well tolerated over 52 weeks and ameliorated lipid abnormalities in hypertriglyceridemic patients with type 2 diabetes.

Matsuba et al. explored the effect of pemafibrate on liver or peripheral insulin resistance, using hyperinsulinemic-euglycemic clamp [96••]. Pemafibrate significantly enhanced the rate of hepatic glucose uptake, thereby improving insulin resistance. In a diet-induced obesity mouse model, pemafibrate suppressed high-fat diet-induced weight gain, reduced plasma glucose and insulin, and increased plasma FGF21. It also upregulated the genes related to thermogenesis and fatty acid β-oxidation and improved obesity-induced metabolic abnormalities [65•]. ABCA1 is involved in efflux of cholesterol and phospholipids from cells to HDL and its deficiency causes Tangier disease characterized by orange tonsils, hepatosplenomegaly, and enhanced atherosclerosis [97]. We reported that patients with Tangier disease showed the progressively increased plasma glucose concentration after oral glucose tolerance test, indicating a type 2 diabetic pattern; however, plasma insulin concentration did not respond well to glucose increase [98]. Calculated insulinogenic index was significantly lower in patients with Tangier disease than in non-diabetic controls. Since ABCA1 is expressed in pancreatic β cells, glucose-stimulated insulin secretion might be impaired in Tangier disease patients with ABCA1 mutations. Pancreatic ABCA1 was shown to play a role in beta cell cholesterol homeostasis, thereby affecting insulin secretion [99].

Pemafibrate treatment increased the mRNA and protein levels of ABCA1 and reduced the cellular cholesterol content in INS-1 cells [100•]. PPARα-specific antagonist GW6471 attenuated pemafibrate-induced ABCA1 expression in INS-1 cells. The promoter activity of ABCA1 was increased with pemafibrate. Glucose-stimulated insulin secretion was ameliorated by pemafibrate in INS-1 cells and isolated mouse islets. Although the expression of ABCA1 was reduced in mice fed a high-fat diet, both ABCA1 protein and mRNA levels were increased with pemafibrate treatment. Pemafibrate improved glucose intolerance induced by a high-fat diet in mice, indicating that pemafibrate may enhance insulin secretion by regulation of ABCA1 expression in β cells.

#### Effects of Pemafibrate on Fatty Liver

Recently, non-alcoholic fatty liver disease (NAFLD) and non-alcoholic steatohepatitis (NASH) have become important diseases in the era of metabolic syndrome and abdominal visceral obesity. PPARα-null mice exhibit phenotypes such as severe fatty liver and steatohepatitis [101] and patients with NASH show a reduced expression of liver PPARα [102]. Therefore, PPARα agonists can be one of the therapeutic agents for NAFLD. Although fibrates were effective for improvement of NAFLD in animals [103–105], their efficacy in clinical settings is not established. The poor clinical outcome data of fibrates can be attributed partly to the adverse reactions such as liver and renal dysfunction, which might have caused reduced efficacy of fibrates.

As demonstrated by previous clinical trials, pemafibrate reduced the levels of serum alanine aminotransferase (ALT), alkaline phosphatase (ALP), γ-glutamyl transpeptidase (γ-GT), and total bilirubin. These effects were markedly observed in patients whose liver function values were higher than the baseline reference values [106], indicating that pemafibrate may be applicable for treating patients with NAFLD/NASH.

In mouse models of NAFLD/NASH, pemafibrate improved liver function and histology. It attenuated fatty liver and ballooning as well as inflammation and fibrosis [107•, 108•]. The mechanisms by which pemafibrate improves NAFLD may involve the upregulation of genes for β-oxidation and lipid transport in and out of the liver and enhancing energy metabolism via induction of uncoupling protein 3 (UCP3) genes. To prove the favorable effects of pemafibrate on NAFLD, a phase 2 study is ongoing in Japan (ClinicalTrials.gov Identifier: NCT03350165).

#### Effects of Pemafibrate on Primary Biliary Cholangitis (PBC)

Fenofibrate [109] and bezafibrate [110] were shown to improve liver function test in patients with PBC. Given the favorable effects of pemafibrate on liver function test values in patients with dyslipidemia, it may also improve liver function in patients with PBC. Before exploring clinical trial, a pharmacokinetic study of pemafibrate for patients with PBC is in progress in Japan (JapicCTI-173728). Recent pilot study has indicated that pemafibrate can improve the liver function tests in patients with PBC, although the number of studied subjects is small [111•].

#### Effect of Pemafibrate on Plasma Fibrinogen Levels

Plasma fibrinogen is known to be linked to thrombosis. Conventional fibrates were reported to inhibit the expression of fibrinogen by activation of PPARα. The decrease in fibrinogen levels is a downstream effect of fibrates [112]. In the BIP Study, a large-scale clinical study on bezafibrate, fibrinogen was shown to be a predictor of mortality [113], indicating that the reduction of fibrinogen levels may be one of the mechanisms by which fibrates reduce ASCVD events. Pemafibrate was also demonstrated to have a stronger fibrinogen-reducing effect than fenofibrate [69].

#### Effect of Pemafibrate on Atherosclerosis and Inflammation

One of the main aims of treatment of dyslipidemia is to prevent ASCVD events. The preventive effects of pemafibrate on atherosclerosis have been reported in animal studies. In human apo E2 knock-in mice fed a high-fat, high-cholesterol diet (western diet) [57], plasma total cholesterol, non-HDL-C, and TG were reduced by administration of pemafibrate, while plasma HDL-C levels were increased. The mRNA expressions of small intestine apoB and liver apoC-III were reduced by pemafibrate. Pemafibrate (0.1 mg/kg body weight) was equivalent to or better than fenofibrate (250 mg/kg body weight). Atherosclerotic lesions area was reduced with 1 mg/kg pemafibrate treatment more markedly than with 250 mg/kg fenofibrate. Expressions of F4/80, VCAM1, and IL6 mRNA at the atherosclerotic lesion were significantly reduced, suggesting anti-inflammatory effects of pemafibrate. It was also administered in LDL receptor-null mice and liver apo C-III levels were also reduced [114, 115]. Neointimal formation and macrophage infiltration were also attenuated, suggesting that pemafibrate reduced CD64-positive cells in monocytes, inhibited M1 polarization in IFNγ-stimulated macrophages, and increased the expression of NcoR1/2, co-repressor of proinflammatory cytokines. In another study [116] using LDL receptor-null mice fed a high-fat, high-cholesterol diet, pemafibrate significantly reduced the lipid deposition area within aortic sinus. The MOMA-2-positive area was reduced by 33% compared to the control, indicating the inhibition of macrophage infiltration into the plaques.

In hyperlipidemic pigs, pemafibrate (30 mg/day) was administered for 35 days with coronary stent indwelled on day 7, and animals were observed for the following 28 days [117]. Neointimal formation was significantly attenuated by 26.3% in the pemafibrate-treated group compared with the control group, and it also inhibited inflammatory cells accumulation. In LDL receptor-deficient pig model [118], balloon failure was induced 2 weeks after pemafibrate administration, and 8 weeks later, both macrophage ratio in the plaques and the mRNA levels of c-Jun, NF-κB, and MMP-9 were significantly reduced in the pemafibrate group compared to the control group, indicating that pemafibrate may have an anti-atherosclerotic and anti-inflammatory effect.

### Characteristic Features of Pemafibrate Distinct from Fibrates

It is important to discriminate SPPARMα, pemafibrate, from conventional fibrates. Table 1 summarizes and compares the effects of pemafibrate and fenofibrate on clinical parameters. Regarding the effects on serum lipids, lipoproteins, and apolipoproteins, pemafibrate 0.4 mg/day showed changes comparable to fenofibrate 200 mg/day. However, marked differences are noted between pemafibrate and fenofibrate with regard to liver function tests, serum creatinine, and FGF21 levels. Especially, ALT, γ-GT, and ALP levels were reduced by ~ 8 U/L, ~ 24 U/L, and ~ 70–80 U/L by pemafibrate, respectively.

**Table 1:** Effects of pemafibrate (SPPARMα) and fenofibrate on clinical parameters

Parameters		Pemafibrate (SPPARMα)			Fenofibrate		
		0.1 mg/day	0.2 mg/day	0.4 mg/day	100 mg/day	106.6 mg/day	200 mg/day
% Change							
TG	1)	− 36.4%	− 42.6%	− 42.7%	− 29.7%		
	2)		− 46.2%	− 45.9%		− 39.7%	
	3)	− 46.3%	− 46.7%	− 51.8%	− 38.3%		− 51.5%
HDL-C	1)	16.5%	16.3%	21.0%	14.3%		
	2)		22.3%	17.4%		17.6%	
	3)	20.9%	21.4%	19.1%	15.2%		24.7%
Non-HDL-C	1)	− 11.8%	− 12.2%	− 10.5%	− 10.1%		
	2)		− 11.1%	− 8.1%		− 11.4%	
	3)	− 5.1%	− 4.0%	− 2.7%	− 2.9%		− 10.7%
LDL-C	1)	8.3%	5.0%	7.4%	5.3%		
	2)		− 6.3%	− 3.5%		− 6.3%	
	3)	13.2%	18.6%	19.3%	14.0%		6.6%
CM-C	1)	− 55.7%	− 67.2%	− 63.4%	− 47.6%		
VLDL-C	1)	− 37.3%	− 43.8%	− 48.4%	− 25.8%		
	3)	− 40.4%	− 44.1%	− 47.1%	− 29.5%		− 47.8%
RemL-C	1)	− 42.8%	− 48.3%	− 50.1%	− 31.8%		
	3)	− 46.8%	− 47.6%	− 50.3%	− 34.5%		− 49.3%
Apo A-I	1)	4.6%	6.0%	8.6%	5.6%		
	2)		18.8%	16.5%		15.0%	
	3)	7.3%	7.9%	6.7%	4.9%		9.2%
Apo A-II	1)	14.4%	21.0%	30.0%	20.1%		
	2)		28.6%	31.7%		22.0%	
	3)	16.5%	21.5%	28.7%	15.8%		30.4%
Apo B	1)	− 8.9%	− 7.8%	− 8.1%	− 5.7%		
	2)		− 8.7%	− 5.6%		− 9.9%	
	3)	0.3%	− 0.4%	3.2%	1.2%		− 7.3%
Apo B-48	1)	− 43.1%	− 55.9%	− 51.2%	− 37.9%		
	3)	− 46.6%	− 51.5%	− 59.0%	− 40.1%		− 51.4%
Apo C-III	1)	− 29.0%	− 34.6%	− 33.4%	− 27.2%		
	3)	− 22.9%	− 31.9%	− 36.3%	− 20.0%		− 33.5%
TG AUC_0 – 8.5 h_	4)			~ − 40%			
Apo B-48 AUC_0 – 8.5 h_	4)			~ − 40%			
RemL-C AUC_0 – 8.5 h_	4)			~ − 45%			
Cholesterol efflux capacity	4)			7.82%			
Change							
Glucose (mmol/L)	1)	− 0.04	− 0.28	− 0.06	− 0.32		
	2)		− 0.2	− 0.1		− 0.1	
	3)	0.9 mg/dL	− 2.0 mg/dL	− 5.7 mg/dL	− 1.2 mg/dL		− 3.3 mg/dL
Insulin (pmol/L)	1)	− 8.58	− 55.50	− 14.52	− 3.45		
	2)		− 4.5	− 7.9		− 1.2	
	3)	− 1.0 μU/mL	− 1.8 μU/mL	− 4.1 μU/mL	− 2.1 μU/mL		− 2.2 μU/mL
HOMA-IR	1)	− 0.33	− 2.65	− 0.50	− 0.38		
	2)		− 0.2	− 0.3		− 0.0	
	3)	− 0.3	− 0.6	− 1.8	− 0.7		− 0.9
Glucose uptake	5)			SGU 19.6%			
ALT (U/L)	1)	− 6.6	− 7.6	− 8.7	− 4.2		
	2)		− 8.3 ^a^	− 4.8 ^a^		2.9 ^a^	
γ-GT (U/L)	1)	− 18.1	− 24.6	− 24.4	0.0		
	2)		− 18.8 ^a^	− 22.9 ^a^		− 3.0 ^a^	
ALP (U/L)	1)	− 52.1	− 66.3	− 68.6	− 48.0		
	2)		− 82.1 ^a^	− 77.7 ^a^		− 46.3 ^a^	
Serum creatinine (mg/dL)	1)	− 0.014	0.013	0.050	0.086		
	2)		0.0 ^a^	0.0 ^a^		0.1 ^a^	
Cystatin C (mg/L)	2)		0 ^a^	0 ^a^		0.1 ^a^	
Homocysteine (nmol/mL)	1)	− 0.08	0.14	1.16	2.21		
	2)		1.3 ^a^	1.0 ^a^		2.2 ^a^	
Fibrinogen (mg/dL)	1)	− 49.2	− 39.7	− 60.1	− 33.4		
	2)		− 43.3 ^a^	− 54.9 ^a^		− 40.3 ^a^	
FGF21 (log [pg/mL])	1)	0.66	0.42	0.78	0.16		

Abbreviations

*SPPARMα* selective peroxisome proliferator-activated receptor α modulator, *TG* triglyceride, *HDL-C* high-density lipoprotein cholesterol, *LDL-C* low-density lipoprotein cholesterol, *CM-C* chylomicron cholesterol, *VLDL-C* very-low-density lipoprotein cholesterol, *RemL-C* remnant lipoprotein cholesterol, *apo* apolipoprotein, *AUC*_*0–8.5 h*_ area under the curve over 8.5 h, *HOMA-IR* homeostasis model assessment for insulin resistance, *SGU* splanchnic glucose uptake, *ALT* alanine aminotransferase, *γ-GT* γ-glutamyl transpeptidase, *ALP* alkaline phosphatase, *FGF21* fibroblast growth factor 21

^a^Calculated by simply subtracting the value at 0 week from that at 24 weeks in K-877-17 Trial

1) Ref [69] [K-877-04 Trial]

2) Ref [81] [K-877-17 Trial]

3) Ref [80] [K-877-09 Trial]

4) Ref [70•] [K-877-11 Trial]

5) Ref [96••] [K-877-19 Trial]

In basic animal and cell biological studies, pemafibrate enhanced more markedly the expressions of ABCA1 and ABCG1 in macrophages and attenuated the proinflammatory genes such as VCAM1, F4/80 (macrophages), and IL6. Table 2 summarizes and compares the effects of pemafibrate and fenofibrate on basic parameters. Distinct differences between pemafibrate and fenofibrate are demonstrated.

**Table 2:** Effects of pemafibrate (SPPARMα) and fenofibrate on basic parameters

Parameters		Pemafibrate (SPPARMα)	Fenofibrate
ABCA1 in human primary macrophages	1)	+563% (at 10 μM)	+ 168% (at 100 μM)
ABCG1 in human primary macrophages	1)	+2093% (at 10 μM)	+ 506% (at 100 μM)
HDL stimulated cholesterol efflux in human primary macrophages	1)	1.9-fold (at 10 μM)	1.6-fold (at 100 μM)
VCAM1 in apoE2KI mice	1)	−32% (at 1 mpk)	NS (at 250 mpk)
F4/80 in apoE2KI mice	1)	− 30% (at 1 mpk)	NS (at 250 mpk)
IL6 in apoE2KI mice	1)	− 40% (at 1 mpk)	NS (at 250 mpk)
Aortic lesion in apoE2KI mice	1)	0.061 mm^2^ (at 0.1 mpk)	0.06 mm^2^ (at 250 mpk)
		0.022 mm^2^ (at 1 mpk)	0.207 mm^2^ (at control)
		0.207 mm^2^ (at control)	
Liver pathology score in amylin liver NASH model	2)	[NAS] 6.2 (at 0.03 mg/kg), 3.9 (at 0.1 mg/kg)	[NAS] 5.7 (at 50 mg/kg)
		[Steatosis] 3 (at 0.03 mg/kg), 1.3 (at 0.1 mg/kg)	[Steatosis] 2.4 (at 50 mg/kg)
		[Lobular inflammation] 1.2 (at 0.03 mg/kg), 1.3 (at 0.1 mg/kg)	[Lobular inflammation] 1.4 (at 50 mg/kg)
		[Hepatocyte ballooning] 2 (at 0.03 mg/kg), 1.3 (at 0.1 mg/kg)	[Hepatocyte ballooning] 1.9 (at 50 mg/kg)
		[Fibrosis] 1.6 (at 0.03 mg/kg), 1.4 (at 0.1 mg/kg)	[Fibrosis] 1.3 (at 50 mg/kg)
Fatty acid oxidation (in the liver of MCD-fed mice)	3)	[Acox1 mRNA] ↑ (at 0.00025%)	[Acox1 mRNA] ↑ (at 0.1%)
		[Cpt1a mRNA] ↑ (at 0.00025%)	[Cpt1a mRNA] ↑ (at 0.1%)
ER stress (in the liver of MCD-fed mice)	3)	[Xbp1s mRNA] ↓ (at 0.00025%)	[Xbp1s mRNA] ↓ (NS, at 0.1%)
LPL activity (male C57BL/6 J mice fed HFD)	4)	↑ (at 0.0005%)	↑ (at 0.05%)
Npc1l1 mRNA in the intestinal epithelial cells (male C57BL/6 J mice fed HFD)	4)	↓ (at 0.0005%)	↓ (at 0.05%)
Mttp mRNA in the intestinal epithelial cells (male C57BL/6 J mice fed HFD)	4)	→ (at 0.0005%)	↑ (at 0.05%)
Hsl in eWAT (male WT mice fed HFD)	5)	↑ (at 0.00033%)	→ (at 0.2%)
Ucp1 in iWAT (male WT mice fed HFD)	5)	↑↑ (at 0.00033%)	↑ (at 0.2%)
Elovl3 in BAT (male WT mice fed HFD)	5)	↑ (at 0.00033%)	↑ (at 0.2%)

Abbreviations

*ABCA1* ATP-binding cassette transporter A1, *ABCG1* ATP-binding cassette transporter G1, *VCAM1* vascular cell adhesion molecule 1, *IL6* interleukin 6, *KI* knock-in, *NASH* non-alcoholic steatohepatitis, *NAS* non-alcoholic fatty liver disease activity score, *MCD* methionine-choline-deficient, *Acox1* acyl-CoA oxidase 1, *Cpt1a* carnitine palmitoyltransferase 1a, *ER* endoplasmic reticulum, *Xbp1s* X-box binding protein 1s, *LPL* lipoprotein lipase, *HFD* high-fat diet, *Npc1l1* niemann-pick c1-like 1, *Mttp* microsomal triglyceride transfer protein, *Hsl* hormone sensitive lipase, *NS* not significant, *eWAT* epididymal white adipose tissue, *Ucp1* uncoupling protein 1, *iWAT* inguinal white adipose tissue, *Elovl3* ELOVL fatty acid elongase 3, *BAT* brown adipose tissue

1) Ref [57]

2) Ref [108•]

3) Ref [107•]

4) Ref [60•]

5) Ref [65•]

### Future Perspectives of SPPARMα, Pemafibrate

#### Pemafibrate for Prevention of CV Events

To explore the effect of pemafibrate on reducing CV events in humans, a large-scale clinical study, PROMINENT (Pemafibrate to Reduce cardiovascular OutcoMes by reducing triglycerides IN diabetic patiENTs) Study (ClinicalTrials.gov Identifier: NCT03071692) [119], is currently ongoing in 24 countries worldwide, including Japan, the USA, the UK, and Russia. It is planned to recruit 10,000 patients with type 2 diabetes who also have high TG and low HDL-C, with LDL-C controlled by drugs such as statins.

#### Pemafibrate for Prevention of Diabetic Microangiopathy

Fibrates were indicated to protect against diabetic microangiopathy in large-scale clinical studies [120]. In the FIELD study, the administration of fenofibrate to patients with type 2 diabetes ameliorated diabetic retinopathy and nephropathy. The number of photocoagulation procedures required for diabetic retinopathy and microalbuminuria were decreased [8, 121]. Thus, pemafibrate was expected to attenuate diabetic microangiopathy. In *db/db* mice, pemafibrate reduced the expression of NOX4 in association with inhibition of PKC activity, by reducing the diacylglycerol content in the kidneys, and also attenuated oxidative stress-induced renal damage [122]. Therefore, pemafibrate may have anti-oxidative and anti-inflammatory functions, thereby possessing favorable effects on diabetic microangiopathy. The effect of pemafibrate on attenuation of progression of diabetic retinopathy should be evaluated in future studies.

Pemafibrate is a novel class of drug distinct from fibrates; thus, it may exhibit novel effects on diabetic complications. Patients with type 2 diabetes often express a reduction of eGFR due to diabetic nephropathy, but renally metabolized fibrates such as fenofibrate, bezafibrate, and clofibrate cannot be used because of an increase in plasma drug concentration. In contrast, pemafibrate is mainly metabolized by the liver and little is excreted into the urine, so it can be used for diabetic patients with nephropathy. Therefore, pemafibrate could be an essential drug for diabetic patients and/or chronic kidney disease (CKD).

Patients with extremely high serum TG levels (> 1000 mg/dL) are known to have an increased risk of acute pancreatitis after an intake of fat-rich foods. Pemafibrate was not administered to patients with such high TG levels in the developmental clinical studies. Two studies in Europe and the US are currently ongoing in patients with severe hypertriglyceridemia (ClinicalTrials.gov Identifier: NCT03011450, NCT03001817). It may be essential to explore whether pemafibrate may also be effective for such patients with severe hypertriglyceridemia in Japan, where fat consumption is much less than in the western countries.

## Conclusions

**Fig. 3:**
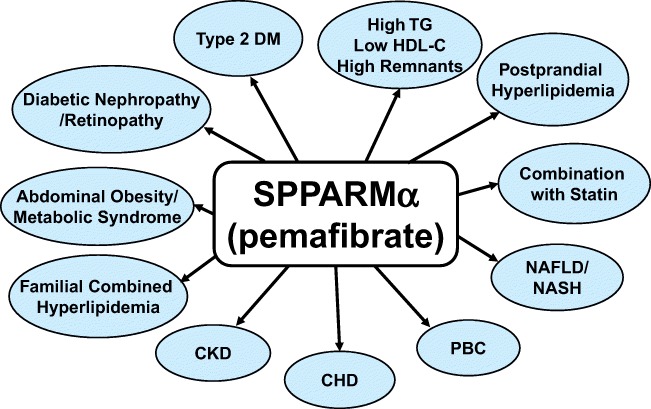
Applications of SPPARMα, pemafibrate, to a variety of metabolic diseases Abbreviation: CKD, chronic kidney disease; CHD, coronary heart disease; HDL-C, high-density lipoprotein cholesterol; NASH, non-alcoholic steatohepatitis; PBC, primary biliary cholangitis; RemL-C, remnant lipoprotein cholesterol; TG, triglycerides

Pemafibrate is the first SPPARMα developed based upon a completely new concept. It has been shown to have a high selectivity for PPARα and possess distinct differences from the conventional fibrates. Pemafibrate can be applied for patients with a variety of metabolic diseases as illustrated in Fig. 3. Pemafibrate is mainly metabolized by the liver and not excreted from the kidneys; therefore, it can be administered to patients with CKD. Pemafibrate has no drug-drug interactions with statins, and its co-administration with any statin is safe. Pemafibrate may have a better risk-benefit balance than the existing fibrates and is considered to be a safer drug for patients with limited response to available fibrates, including patients taking statins, and patients with compromised kidney functions or hepatic steatosis. The first SPPARMα, pemafibrate, developed in Japan ahead of the rest of the world, is expected to have a better efficacy than fibrates as a new therapeutic option for dyslipidemia as well as diabetic complications and liver diseases.

## References

[1] Oliver M (2012). The clofibrate saga: a retrospective commentary. Br J Clin Pharmacol.

[2] Staels B, Dallongeville J, Auwerx J, Schoonjans K, Leitersdorf E, Fruchart JC (1998). Mechanism of action of fibrates on lipid and lipoprotein metabolism. Circulation..

[3] Fruchart JC, Duriez P, Staels B (1999). Peroxisome proliferator-activated receptor-alpha activators regulate genes governing lipoprotein metabolism, vascular inflammation and atherosclerosis. Curr Opin Lipidol.

[4] Frick MH, Elo O, Haapa K, Heinonen OP, Heinsalmi P, Helo P (1987). Helsinki heart study: primary-prevention trial with gemfibrozil in middle-aged men with dyslipidemia. Safety of treatment, changes in risk factors, and incidence of coronary heart disease. N Engl J Med.

[5] Rubins HB, Robins SJ, Collins D, Fye CL, Anderson JW, Elam MB (1999). Gemfibrozil for the secondary prevention of coronary heart disease in men with low levels of high-density lipoprotein cholesterol. Veterans Affairs High-Density Lipoprotein Cholesterol Intervention Trial Study Group. N Engl J Med.

[6] Jacobson TA (2009). Myopathy with statin-fibrate combination therapy: clinical considerations. Nat Rev Endocrinol.

[7] Bezafibrate Infarction Prevention (BIP) Study Group, Secondary prevention by raising HDL cholesterol and reducing triglycerides in patients with coronary artery disease (2000). The Bezafibrate Infarction Prevention (BIP) Study. Circulation.

[8] Keech A, Simes RJ, Barter P, Best J, Scott R, Taskinen MR (2005). Effects of long-term fenofibrate therapy on cardiovascular events in 9795 people with type 2 diabetes mellitus (the FIELD study): randomised controlled trial. Lancet..

[9] Ginsberg HN, Elam MB, Lovato LC, Crouse JR, Leiter LA, Linz P (2010). Effects of combination lipid therapy in type 2 diabetes mellitus. N Engl J Med.

[10] Jun M, Foote C, Lv J, Neal B, Patel A, Nicholls SJ (2010). Effects of fibrates on cardiovascular outcomes: a systematic review and meta-analysis. Lancet..

[11] Ip CK, Jin DM, Gao JJ, Meng Z, Meng J, Tan Z (2015). Effects of add-on lipid-modifying therapy on top of background statin treatment on major cardiovascular events: a meta-analysis of randomized controlled trials. Int J Cardiol.

[12] Sacks FM, Carey VJ, Fruchart JC (2010). Combination lipid therapy in type 2 diabetes. N Engl J Med.

[13] Elam MB, Ginsberg HN, Lovato LC, Corson M, Largay J, Leiter LA (2017). ACCORDION Study investigators: association of fenofibrate therapy with long-term cardiovascular risk in statin-treated patients with type 2 diabetes. JAMA Cardiol.

[14] Arbel Y, Klempfner R, Erez A, Goldenberg I, Benzekry S, Shlomo N (2016). BIP study group: Bezafibrate for the treatment of dyslipidemia in patients with coronary artery disease: 20-year mortality follow-up of the BIP randomized control trial. Cardiovasc Diabetol.

[15] Wang D, Liu B, Tao W, Hao Z, Liu M (2015). Fibrates for secondary prevention of cardiovascular disease and stroke. Cochrane Database Syst Rev.

[16] Jakob T, Nordmann AJ, Schandelmaier S, Ferreira-González I, Briel M (2016). Fibrates for primary prevention of cardiovascular disease events. Cochrane Database Syst Rev.

[17] Baigent C, Keech A, Kearney PM, Blackwell L, Buck G, Pollicino C (2005). Efficacy and safety of cholesterol-lowering treatment: prospective meta-analysis of data from 90,056 participants in 14 randomised trials of statins. Lancet..

[18] Baigent C, Blackwell L, Emberson J, Holland LE, Reith C, Bhala N (2010). Efficacy and safety of more intensive lowering of LDL cholesterol: a meta-analysis of data from 170,000 participants in 26 randomised trials. Lancet..

[19] Fulcher J, O'Connell R, Voysey M, Emberson J, Blackwell L, Mihaylova B (2015). Efficacy and safety of LDL-lowering therapy among men and women: meta-analysis of individual data from 174,000 participants in 27 randomised trials. Lancet..

[20] Ferri N, Corsini A, Sirtori C, Ruscica M (2017). PPAR-α agonists are still on the rise: an update on clinical and experimental findings. Expert Opin Investig Drugs.

[21] Issemann I, Green S (1990). Activation of a member of the steroid hormone receptor superfamily by peroxisome proliferators. Nature..

[22] Dietz M, Mohr P, Kuhn B, Maerki HP, Hartman P, Ruf A (2012). Comparative molecular profiling of the PPARalpha/gamma activator aleglitazar: PPAR selectivity, activity and interaction with cofactors. Chem Med Chem.

[23] Fruchart JC, Staels B, Duriez P (2001). PPARS, metabolic disease and atherosclerosis. Pharmacol Res.

[24] Fruchart JC, Duriez P, Staels B (1999). Molecular mechanism of action of the fibrates. J Soc Biol.

[25] Fruchart JC, Duriez P (2006). Mode of action of fibrates in the regulation of triglyceride and HDL-cholesterol metabolism. Drugs Today (Barc).

[26] Gross B, Pawlak M, Lefebvre P, Staels B (2017). PPARs in obesity-induced T2DM, dyslipidaemia and NAFLD. Nat Rev Endocrinol.

[27] Delerive P, De Bosscher K, Besnard S, Vanden Berghe W, Peters JM, Gonzalez FJ (1999). Peroxisome proliferator-activated receptor alpha negatively regulates the vascular inflammatory gene response by negative cross-talk with transcription factors NF-kappaB and AP-1. J Biol Chem.

[28] Kertsen S (2014). Integrated physiology and systems biology of PPARα. Mol Metab.

[29] Fruchart JC (2009). Peroxisome proliferator-activated receptor-alpha (PPARalpha): at the crossroads of obesity, diabetes and cardiovascular disease. Atherosclerosis..

[30] Lefebvre P, Chinetti G, Fruchart JC, Staels B (2006). Sorting out the roles of PPAR alpha in energy metabolism and vascular homeostasis. J Clin Invest.

[31] Schoonjans P-OJ, Lefebvre AM, Heyman RA, Briggs M, Deeb S (1996). PPARalpha and PPARgamma activators direct a distinct tissue-specific transcriptional response via a PPRE in the lipoprotein lipase gene. EMBO J.

[32] Prieur X, Coste H, Rodriguez JC (2003). The human apolipoprotein AV gene is regulated by peroxisome proliferator-activated receptor-alpha and contains a novel farnesoid X-activated receptor response element. J Biol Chem.

[33] Huang XS, Zhao SP, Bai L, Hu M, Zhao W, Zhang Q (2009). Atorvastatin and fenofibrate increase apolipoprotein AV and decrease triglycerides by up-regulating peroxisome proliferator-activated receptor-alpha. Br J Pharmacol.

[34] Staels B, Vu-Dac N, Kosykh VA, Saladin R, Fruchart JC, Dallongeville J (1995). Fibrates downregulate apolipoprotein C-III expression independent of induction of peroxisomal acyl coenzyme A oxidase. A potential mechanism for the hypolipidemic action of fibrates. J Clin Invest.

[35] Martin G, Schoonjans K, Lefebvre AM, Staels B, Auwerx J (1997). Coordinate regulation of the expression of the fatty acid transport protein and acyl-CoA synthetase genes by PPARalpha and PPARgamma activators. J Biol Chem.

[36] Tokuno A, Hirano T, Hayashi T, Mori Y, Yamamoto T, Nagashima M (2007). The effects of statin and fibrate on lowering small dense LDL- cholesterol in hyperlipidemic patients with type 2 diabetes. J Atheroscler Thromb.

[37] Hirano T (2018). Pathophysiology of diabetic dyslipidemia. J Atheroscler Thromb.

[38] Duval C, Müller M, Kersten S (2007). PPARalpha and dyslipidemia. Biochim Biophys Acta.

[39] Goldberg IJ (1996). Lipoprotein lipase and lipolysis: central roles in lipoprotein metabolism and atherogenesis. J Lipid Res.

[40] Chinetti G, Lestavel S, Bocher V, Remaley AT, Neve B, Torra IP (2001). PPAR-alpha and PPAR-gamma activators induce cholesterol removal from human macrophage foam cells through stimulation of the ABCA1 pathway. Nat Med.

[41] Chinetti G, Gbaguidi FG, Griglio S, Mallat Z, Antonucci M, Poulain P (2000). CLA-1/SR-BI is expressed in atherosclerotic lesion macrophages and regulated by activators of peroxisome proliferator-activated receptors. Circulation.

[42] Perreault L, Bergman BC, Hunerdosse DM, Howard DJ, Eckel RH (2011). Fenofibrate administration does not affect muscle triglyceride concentration or insulin sensitivity in humans. Metabolism..

[43] Fruchart JC (2013). Selective peroxisome proliferator-activated receptor alpha modulators (SPPARMalpha): the next generation of peroxisome proliferator-activated receptor alpha-agonists. Cardiovasc Diabetol.

[44] Fruchart JC (2017). Pemafibrate (K-877), a novel selective peroxisome proliferator-activated receptor alpha modulator for management of atherogenic dyslipidaemia. Cardiovasc Diabetol.

[45] Shelly W, Draper MW, Krishnan V, Wong M, Jaffe RB (2008). Selective estrogen receptor modulators: an update on recent clinical findings. Obstet Gynecol Surv.

[46] Yamazaki Y, Abe K, Toma T, Nishikawa M, Ozawa H, Okuda A (2007). Design and synthesis of highly potent and selective human peroxisome proliferator-activated receptor alpha agonists. Bioorg Med Chem Lett.

[47] •• Yamamoto Y, Takei K, Arulmozhiraja S, Sladek V, Matsuo N, Han SI, et al. Molecular association model of PPARalpha and its new specific and efficient ligand, pemafibrate: Structural basis for SPPARMalpha. Biochem Biophys Res Commun. 2018;499(2):239–45 **This paper computationally constructed the structure of human PPARα in a complex with pemafibrate in comparison with fenofibrate. The binding of pemafibrate to human PPARα was markedly different from that of fenofibrate.** 10.1016/j.bbrc.2018.03.135 29567478

[48] Chinetti-Gbaguidi G, Fruchart JC, Staels B. Role of the PPAR family of nuclear receptors in the regulation of metabolic and cardiovascular homeostasis: new approaches to therapy. Curr Opin Pharmacol. 2005;5(2):177–83. 10.1016/j.coph.2004.11.004 15780828

[49] Takizawa T, Inokuchi Y, Goto S, Yoshinaka Y, Abe K, Inoue K (2013). Abstract 12867: The mechanism of K-877, a highly potent and selective pparalpha modulator, on regulation of synthesis, secretion and metabolism of triglycerides and cholesterol. Circulation.

[50] Willson TM, Brown PJ, Sternbach DD, Henke BR (2000). The PPARs: from orphan receptors to drug discovery. J Med Chem.

[51] Raza-Iqbal S, Tanaka T, Anai M, Inagaki T, Matsumura Y, Ikeda K (2015). Transcriptome analysis of K-877 (a novel selective PPARalpha modulator (SPPARMalpha))-regulated genes in primary human hepatocytes and the mouse liver. J Atheroscler Thromb.

[52] Kinoshita M, Yokote K, Arai H, Iida M, Ishigaki Y, Ishibashi S (2018). Japan atherosclerosis society (JAS) guidelines for prevention of atherosclerotic cardiovascular diseases 2017. J Atheroscler Thromb.

[53] Fruchart JC, Santos RD, Aguilar-Salinas C, Aikawa M, Al Rasadi K, Amarenco P (2019). The selective peroxisome proliferator-activated receptor alpha modulator (SPPARMα) paradigm: conceptual framework and therapeutic potential: A consensus statement from the International Atherosclerosis Society (IAS) and the Residual Risk Reduction Initiative (R3i) Foundation. Cardiovasc Diabetol.

[54] Ogawa SI, Tsunenari Y, Kawai H, Yamazaki H (2019). Pharmacokinetics and metabolism of pemafibrate, a novel selective peroxisome proliferator-activated receptor-alpha modulator, in rats and monkeys. Biopharm Drug Dispos.

[55] Takahashi S (2017). Triglyceride rich lipoprotein-LPL-VLDL receptor and Lp(a)- VLDL receptor pathways for macrophage foam cell formation. J Atheroscler Thromb.

[56] Gao Y, Shen W, Lu B, Zhang Q, Hu Y, Chen Y (2014). Upregulation of hepatic VLDLR via PPARα is required for the triglyceride-lowering effect of fenofibrate. J Lipid Res.

[57] Hennuyer N, Duplan I, Paquet C, Vanhoutte J, Woitrain E, Touche V (2016). The novel selective PPARα modulator (SPPARMα) pemafibrate improves dyslipidemia, enhances reverse cholesterol transport and decreases inflammation and atherosclerosis. Atherosclerosis..

[58] Iwata H, Murakami K, Ricchiuto P, Singh S, Mojcher AC, Libby P (2013). K-877, A novel PPAR-alpha selective agonist, suppresses macrophage activation and arterial lesion formation. Circulation.

[59] Iwata H, Murakami K, Ricchiuto P, Singh S, Libby P, Aikawa E (2014). The novel PPARα selective agonist K-877 suppresses pro-inflammatory pathways and experimental arterial lesion formation. Circ Res.

[60] Sairyo M, Kobayashi T, Masuda D, Kanno K, Zhu Y, Okada T (2018). A novel selective PPARα modulator (SPPARMα), K-877 (pemafibrate), attenuates postprandial hypertriglyceridemia in mice. J Atheroscler Thromb.

[61] Takei K, Nakagawa Y, Wang Y, Han SI, Satoh A, Sekiya M (2017). Effects of K-877, a novel selective PPARalpha modulator, on small intestine contribute to the amelioration of hyperlipidemia in low-density lipoprotein receptor knockout mice. J Pharmacol Sci.

[62] Fisher FM, Chui PC, Nasser IA, Popov Y, Cunniff JC, Lundasen T (2014). Fibroblast growth factor 21 limits lipotoxicity by promoting hepatic fatty acid activation in mice on methionine and choline-deficient diets. Gastroenterology..

[63] Liu J, Xu Y, Hu Y, Wang G (2015). The role of fibroblast growth factor 21 in the pathogenesis of non-alcoholic fatty liver disease and implications for therapy. Metabolism..

[64] Schlein C, Talukdar S, Heine M, Fischer AW, Krott LM, Nilsson SK (2016). FGF21 lowers plasma triglycerides by accelerating lipoprotein catabolism in white and brown adipose tissues. Cell Metab.

[65] Araki M, Nakagawa Y, Oishi A, Han SI, Wang Y, Kumagai K (2018). The peroxisome proliferator-activated receptor alpha (PPARalpha) agonist pemafibrate protects against diet-induced obesity in mice. Int J Mol Sci.

[66] Vu-Dac N, Gervois P, Jakel H, Nowak M, Bauge E, Dehondt H (2003). Apolipoprotein A5, a crucial determinant of plasma triglyceride levels, is highly responsive to peroxisome proliferator-activated receptor alpha activators. J Biol Chem.

[67] Fujioka Y, Ishikawa Y (2009). Remnant lipoproteins as strong key particles to atherogenesis. J Atheroscler Thromb.

[68] Masuda D, Yamashita S (2017). Postprandial hyperlipidemia and remnant lipoproteins. J Atheroscler Thromb.

[69] Ishibashi S, Yamashita S, Arai H, Araki E, Yokote K, Suganami H (2016). Effects of K-877, a novel selective PPARalpha modulator (SPPARMalpha), in dyslipidaemic patients: a randomized, double blind, active- and placebo-controlled, phase 2 trial. Atherosclerosis..

[70] Yamashita S, Arai H, Yokote K, Araki E, Suganami H, Ishibashi S (2018). Effects of pemafibrate (K-877) on cholesterol efflux capacity and postprandial hyperlipidemia in patients with atherogenic dyslipidemia. J Clin Lipidol.

[71] Araki E, Yamashita S, Arai H, Yokote K, Satoh J, Inoguchi T (2018). Effects of pemafibrate, a novel selective PPARalpha modulator, on lipid and glucose metabolism in patients with type 2 diabetes and hypertriglyceridemia: A randomized, double-blind, placebo-controlled, phase 3 trial. Diabetes Care.

[72] Okazaki M, Yamashita S (2016). Recent advances in analytical methods on lipoprotein subclasses: calculation of particle numbers from lipid levels by gel permeation HPLC using "spherical particle model". J Oleo Sci.

[73] Yamashita S, Okazaki M, Okada T, Masuda D, Arai H, Yokote K (2018). Effects of selective PPAR alpha modulator K-877 on particle numbers of lipoprotein subclasses in dyslipidemic patients: Analysis by GP-HPLC and NMR Lipoprofile 2 and 3. Atherosclerosis Supplements.

[74] Khera AV, Cuchel M, de la Llera-Moya M, Rodrigues A, Burke MF, Jafri K (2011). Cholesterol efflux capacity, high-density lipoprotein function, and atherosclerosis. N Engl J Med.

[75] Rohatgi A, Khera A, Berry JD, Givens EG, Ayers CR, Wedin KE (2014). HDL cholesterol efflux capacity and incident cardiovascular events. N Engl J Med.

[76] Du XM, Kim MJ, Hou L, Le Goff W, Chapman MJ, Van Eck M (2015). HDL particle size is a critical determinant of ABCA1-mediated macrophage cellular cholesterol export. Circ Res.

[77] Shang W, Yu X, Wang H, Chen T, Fang Y, Yang X (2015). Fibroblast growth factor 21 enhances cholesterol efflux in THP-1 macrophage-derived foam cells. Mol Med Rep.

[78] Hounslow N, Suganami H, Nakamura M (2018). Pemafibrate minimally affected the systemic exposure of statins, and vice versa, in healthy male volunteers. Atherosclerosis Supplements.

[79] Arai H, Yamashita S, Yokote K, Araki E, Suganami H, Ishibashi S (2017). Efficacy and safety of K-877, a novel selective peroxisome proliferator-activated receptor alpha modulator (SPPARMalpha), in combination with statin treatment: two randomised, double-blind, placebo-controlled clinical trials in patients with dyslipidaemia. Atherosclerosis.

[80] Arai H, Yamashita S, Yokote K, Araki E, Suganami H, Ishibashi S (2018). Efficacy and safety of pemafibrate versus fenofibrate in patients with high triglyceride and low HDL cholesterol levels: A multicenter, placebo-controlled, double-blind, randomized trial. J Atheroscler Thromb.

[81] Ishibashi S, Arai H, Yokote K, Araki E, Suganami H, Yamashita S (2018). Efficacy and safety of pemafibrate (K-877), a selective peroxisome proliferator-activated receptor alpha modulator, in patients with dyslipidemia: Results from a 24-week, randomized, double blind, active-controlled, phase 3 trial. J Clin Lipidol.

[82] Sahebkar A, Simental-Mendia LE, Pirro M, Montecucco F, Carbone F, Banach M (2018). Impact of fibrates on circulating cystatin C levels: a systematic review and meta-analysis of clinical trials. Ann Med.

[83] Ncube V, Starkey B, Wang T (2012). Effect of fenofibrate treatment for hyperlipidaemia on serum creatinine and cystatin C. Ann Clin Biochem.

[84] Davidson MH, Armani A, McKenney JM, Jacobson TA (2007). Safety considerations with fibrate therapy. Am J Cardiol.

[85] Edgar AD, Tomkiewicz C, Costet P, Legendre C, Aggerbeck M, Bouguet J (1998). Fenofibrate modifies transaminase gene expression via a peroxisome proliferator activated receptor alpha-dependent pathway. Toxicol Lett.

[86] Ida S, Kaneko R, Murata K (2019). Efficacy and safety of pemafibrate administration in patients with dyslipidemia: a systematic review and meta-analysis. Cardiovasc Diabetol..

[87] Barter P (2014). Lipoprotein metabolism and CKD: overview. Clin Exp Nephrol.

[88] Wanner C, Krane V, März W, Olschewski M, Mann JF, Ruf G (2005). German Diabetes and Dialysis Study Investigators: atorvastatin in patients with type 2 diabetes mellitus undergoing hemodialysis. N Engl J Med.

[89] Fellström BC, Jardine AG, Schmieder RE, Holdaas H, Bannister K, Beutler J (2009). Rosuvastatin and cardiovascular events in patients undergoing hemodialysis. N Engl J Med.

[90] Cannon CP, Blazing MA, Giugliano RP, McCagg A, White JA, Theroux P, et al; IMPROVE-IT Investigators: Ezetimibe added to statin therapy after acute coronary syndromes. N Engl J Med 2015;372(25):2387–2397. 10.1056/NEJMoa1410489 26039521

[91] Hounslow N, Mair S, Suganami H, Nakamura M (2018). Pemafibrate has high bioavailability and is principally excreted via the liver. Atherosclerosis Supplements.

[92] Blair HA (2017). Pemafibrate: First Global Approval. Drugs..

[93] Hosford D, Gordon G, Suganami H, Nakamura M (2018). The plasma concentration and pharmacokinetic parameters of pemafibrate did not increase in a creatinine clearance-dependent manner. Atherosclerosis Supplements.

[94] Yokote K, Yamashita S, Arai H, Araki E, Suganami H, Ishibashi S (2019). Long-term efficacy and safety of pemafibrate, a novel selective peroxisome proliferator-activated receptor-α modulator (SPPARMα), in dyslipidemic patients with renal impairment. Int J Mol Sci.

[95] Araki E, Yamashita S, Arai H, Yokote K, Satoh J, Inoguchi T (2019). Efficacy and safety of pemafibrate in people with type 2 diabetes and elevated triglyceride levels: 52-week data from the PROVIDE study. Diabetes Obes Metab.

[96] •• Matsuba I, Matsuba R, Ishibashi S, Yamashita S, Arai H, Yokote K, et al: Effects of a novel selective peroxisome proliferator-activated receptor-alpha modulator, pemafibrate, on hepatic and peripheral glucose uptake in patients with hypertriglyceridemia and insulin resistance. J Diabetes Investig. 2018;9(6):1323–1332. **By using a hyperinsulinemic-euglycemic clamp technique, splanchnic and peripheral glucose uptake were investigated in patients with hypertriglyceridemia and insulin resistance. Pemafibrate increased splanchnic glucose uptake from baseline and improved insulin resistance.** 10.1111/jdi.12845 PMC6215940 29603684

[97] Muratsu J, Koseki M, Masuda D, Yasuga Y, Tomoyama S, Ataka K (2018). Accelerated atherogenicity in Tangier disease. J Atheroscler Thromb.

[98] Koseki M, Matsuyama A, Nakatani K, Inagaki M, Nakaoka H, Kawase R (2009). Impaired insulin secretion in four Tangier disease patients with ABCA1 mutations. J Atheroscler Thromb.

[99] Brunham LR, Kruit JK, Verchere CB, Hayden MR (2008). Cholesterol in islet dysfunction and type 2 diabetes. J Clin Invest.

[100] Dong T, Lyu J, Imachi H, Kobayashi T, Fukunaga K, Sato S (2018). Selective peroxisome proliferator-activated receptor-α modulator K-877 regulates the expression of ATP-binding cassette transporter A1 in pancreatic beta cells. Eur J Pharmacol.

[101] Ip E, Farrell GC, Robertson G, Hall P, Kirsch R, Leclercq I (2003). Central role of PPARalpha-dependent hepatic lipid turnover in dietary steatohepatitis in mice. Hepatology..

[102] Fujita K, Nozaki Y, Wada K, Yoneda M, Fujimoto Y, Fujitake M (2009). Dysfunctional very-low-density lipoprotein synthesis and release is a key factor in nonalcoholic steatohepatitis pathogenesis. Hepatology..

[103] Ip E, Farrell G, Hall P, Robertson G, Leclercq I (2004). Administration of the potent PPARalpha agonist, Wy-14,643, reverses nutritional fibrosis and steatohepatitis in mice. Hepatology..

[104] Shiri-Sverdlov R, Wouters K, van Gorp PJ, Gijbels MJ, Noel B, Buffat L (2006). Early diet-induced non-alcoholic steatohepatitis in APOE2 knock-in mice and its prevention by fibrates. J Hepatol.

[105] Larter CZ, Yeh MM, Van Rooyen DM, Brooling J, Ghatora K, Farrell GC (2012). Peroxisome proliferator-activated receptor-alpha agonist, Wy 14,643, improves metabolic indices, steatosis and ballooning in diabetic mice with non-alcoholic steatohepatitis. J Gastroenterol Hepatol.

[106] Yokote K, Yamashita S, Arai H, Araki E, Suganami H, Ishibashi S (2018). A pooled analysis of pemafibrate phase II/III clinical trials indicated significant improvement in glycemic and liver function-related parameters. Atherosclerosis Supplements.

[107] Takei K, Han SI, Murayama Y, Satoh A, Oikawa F, Ohno H (2017). Selective peroxisome proliferator-activated receptor-alpha modulator K-877 efficiently activates the peroxisome proliferator-activated receptor-alpha pathway and improves lipid metabolism in mice. J Diabetes Investig.

[108] Honda Y, Kessoku T, Ogawa Y, Tomeno W, Imajo K, Fujita K (2017). Pemafibrate, a novel selective peroxisome proliferator-activated receptor alpha modulator, improves the pathogenesis in a rodent model of nonalcoholic steatohepatitis. Sci Rep.

[109] Suraweera D, Rahal H, Jimenez M, Viramontes M, Choi G, Saab S (2017). Treatment of primary biliary cholangitis ursodeoxycholic acid non-responders: a systematic review. Liver Int.

[110] Corpechot C, Chazouillères O, Rousseau A, Le Gruyer A, Habersetzer F, Mathurin P (2018). A placebo-controlled trial of bezafibrate in primary biliary cholangitis. N Engl J Med.

[111] • Joshita S, Umemura T, Yamashita Y, Sugiura A, Yamazaki T, Fujimori N, et al. Biochemical and plasma lipids responses to pemafibrate in patients with primary biliary cholangitis. Hepatol Res. 2019. 10.1111/hepr.13361 [Epub ahead of print]). **This is a pilot study showing that pemafibrate might be another option for primary biliary cholangitis (PBC) patients with an incomplete response to UDCA therapy.** 10.1111/hepr.13361 31077509

[112] Kockx M, Gervois PP, Poulain P, Derudas B, Peters JM, Gonzalez FJ (1999). Fibrates suppress fibrinogen gene expression in rodents via activation of the peroxisome proliferator-activated receptor-alpha. Blood..

[113] Benderly M, Graff E, Reicher-Reiss H, Behar S, Brunner D, Goldbourt U (1996). Fibrinogen is a predictor of mortality in coronary heart disease patients. The Bezafibrate Infarction Prevention (BIP) Study Group. Arterioscler Thromb Vasc Biol.

[114] Iwata H, Murakami K, Ricchiuto P, Singh S, Libby P, Aikawa E (2014). The novel PPARα selective agonist K-877 suppresses pro-inflammatory pathways and experimental arterial lesion formation. Circ Res.

[115] Iwata H, Murakami K, Ricchiuto P, Singh S, Mojcher A, Libby P (2015). Selective PPAR alpha agonist, K-877 suppresses macrophage activation and experimental arterial lesion formation. Eur Heart J.

[116] Takizawa T, Goto S, Inokuchi Y, Miyata S, Yoshinaka Y, Yamazaki H (2015). Pharmacological effects of K-877, a potent and selective PPAR alpha modulator (SPPARM alpha)-Controlling the plasma HDL-C and triglycerides, and prevention of atherosclerosis in experimental animals. Eur Heart J..

[117] Iwata H, Osborn E, Ughi G, Murakami K, Goettsch C, Hutcheson J (2016). A highly selective PPARA agonist K-877 suppresses neointima formation following coronary stenting in swine. J Am Coll Cardiol.

[118] Konishi H, Miyauchi K, Wada H, Naito R, Tsuboi S, Ogita M (2017). Abstract 15623: effect of pemafibrate (K-877), a novel selective peroxisome proliferator-activated receptor α modulator (SPPARMa), in atherosclerosis model using low density lipoprotein receptor knock-out swine with balloon injury. Circulation.

[119] Pradhan AD, Paynter NP, Everett BM, Glynn RJ, Amarenco P, Elam M (2018). Rationale and design of the pemafibrate to reduce cardiovascular outcomes by reducing triglycerides in patients with diabetes (PROMINENT) study. Am Heart J.

[120] Hiukka A, Maranghi M, Matikainen N, Taskinen MR (2010). PPARalpha: an emerging therapeutic target in diabetic microvascular damage. Nat Rev Endocrinol..

[121] Keech AC, Mitchell P, Summanen PA, O'Day J, Davis TM, Moffitt MS (2007). Effect of fenofibrate on the need for laser treatment for diabetic retinopathy (FIELD study): a randomised controlled trial. Lancet.

[122] Maki T, Maeda Y, Sonoda N, Makimura H, Kimura S, Maeno S (2017). Renoprotective effect of a novel selective PPARalpha modulator K-877 in db/db mice: a role of diacylglycerol-protein kinase C-NAD(P) H oxidase pathway. Metabolism..

